# 3 Tesla is the preferred field strength for perfusion imaging in coronary artery disease – a comparison to 1.5 Tesla and fractional flow reserve

**DOI:** 10.1186/1532-429X-15-S1-W4

**Published:** 2013-01-30

**Authors:** P Bernhardt, T Walcher, D Buckert, W Rottbauer, V Rasche

**Affiliations:** 1 University of Ulm, Ulm, Germany

## Background

Adenosine perfusion imaging at 1.5 Tesla (T) has been shown to yield good diagnostic values in comparison to quantitative coronary angiography. Perfusion imaging at 3 T has the potential benefit of higher contrast- and signal-to-noise ratios. However, little is known about diagnostic performance of 3 T perfusion imaging in comparison to 1.5 T and in comparison to invasive measurement of fractional flow reserve (FFR). We sought to evaluate visual and quantitative assessment of adenosine perfusion at 3 T in comparison to 1.5 T and to coronary angiography in patients presenting with suspected coronary artery disease (CAD).

## Methods

86 consecutive patients with suspected CAD were enrolled in this study. All patients underwent adenosine perfusion at 3 T (Achieva, Philips Medical Systems) and 1.5 T (Intera, Philips Medical Systems) within 72 hours with subsequent coronary angiography within 72 hours. Two independent patient groups were formed. In group 1 1 (N=52) quantitative coronary analysis (QCA) was compared to qualitative perfusion MRI imaging. Group 2 (N=34) underwent FFR measurement in all three major coronary arteries in comparison to quantitative myocardial perfusion reserve (MPR).

## Results

In both groups a significant (p<0.05) higher sensitivity and specificity (group 1: 0.88 vs 0.80 and 0.96 vs. 0.89, p<0.01; group 2 0.91 vs. 0.62 and 1.0 vs. 0.77, p<0.001) was found for 3 T when compared to 1.5 T.

## Conclusions

3 T appears to be the superior field strength for visual or quantitative analysis of myocardial perfusion in CAD as shown in our two consecutive patient groups.

